# Mechanisms of Side Branching and Tip Splitting in a Model of Branching Morphogenesis

**DOI:** 10.1371/journal.pone.0102718

**Published:** 2014-07-22

**Authors:** Yina Guo, Mingzhu Sun, Alan Garfinkel, Xin Zhao

**Affiliations:** 1 Institute of Robotics and Automatic Information Systems, Nankai University, Tianjin, China; 2 Department of Medicine, University of California Los Angeles, Los Angeles, California, United States of America; 3 Department of Integrative Biology and Physiology, University of California Los Angeles, Los Angeles, California, United States of America; 4 Tianjin Key Laboratory of Intelligent Robotics, Tianjin, China; 5 State Key Laboratory of Robotics, Shenyang, China; University of Southampton, United Kingdom

## Abstract

Recent experimental work in lung morphogenesis has described an elegant pattern of branching phenomena. Two primary forms of branching have been identified: side branching and tip splitting. In our previous study of lung branching morphogenesis, we used a 4 variable partial differential equation (PDE), due to Meinhardt, as our mathematical model to describe the reaction and diffusion of morphogens creating those branched patterns. By altering key parameters in the model, we were able to reproduce all the branching styles and the switch between branching modes. Here, we attempt to explain the branching phenomena described above, as growing out of two fundamental instabilities, one in the longitudinal (growth) direction and the other in the transverse direction. We begin by decoupling the original branching process into two semi-independent sub-processes, 1) a classic activator/inhibitor system along the growing stalk, and 2) the spatial growth of the stalk. We then reduced the full branching model into an activator/inhibitor model that embeds growth of the stalk as a controllable parameter, to explore the mechanisms that determine different branching patterns. We found that, in this model, 1) side branching results from a pattern-formation instability of the activator/inhibitor subsystem in the longitudinal direction. This instability is far from equilibrium, requiring a large inhomogeneity in the initial conditions. It successively creates periodic activator peaks along the growing stalk, each of which later on migrates out and forms a side branch; 2) tip splitting is due to a Turing-style instability along the transversal direction, that creates the spatial splitting of the activator peak into 2 simultaneously-formed peaks at the growing tip, the occurrence of which requires the widening of the growing stalk. Tip splitting is abolished when transversal stalk widening is prevented; 3) when both instabilities are satisfied, tip bifurcation occurs together with side branching.

## Introduction

Recent experimental work in lung morphogenesis has described an elegant pattern of branching phenomena [1]. Two primary forms of branching have been identified: side branching and tip splitting. In the lung, these occur in sequence: first, side branching creates the primary stalks; then, there is a change of mode to tip splitting. These phenomena have been hypothesized to be under genetic controls [1], [2], however, how genes could possibly act to produce these patterns is still not clear.

In a previous study of lung branching morphogenesis [3], we used a 4 variable partial differential equation (PDE), due to Meinhardt [4], as our mathematical model to describe the reaction and diffusion of morphogens creating branched lung development. When we simulated this model in 2D and 3D, we were able to successfully reproduce the cascades of branching styles that have been observed in the lung, including side branching and tip splitting [1], [3]. Different branching modes can be produced by altering key parameters. However, to say ‘a change in parameter X produces phenomenon Y’, while interesting, does not give us a real mechanism.

Here, we attempt to explain these phenomena as growing out of two fundamental instabilities, one in the longitudinal (growth) direction and the other in the transverse direction.

We begin by decoupling the full Meinhardt model into two semi-independent sub-processes: 1) a classic activator-inhibitor system on the growing stalk, and 2) spatial extension of the stalk. We then used a reduced activator-inhibitor model that embeds growth of the stalk as our tool to explore these mechanisms.

We found that, in this model, side branching and tip bifurcation occur due to distinct mechanisms. They do not contradict each other, and can occur separately or together. The two distinct instabilities occur in different directions, and their interaction determines the final branched pattern.

The longitudinal instability produces periodic insertion of activator peaks along the growing Y-stalk, each of which later on migrates out and forms a side branch. It does not require the existence of a temporal oscillator, and is therefore distinct from the mechanisms proposed in other studies of side branching, for example, in vertebrate segmentation and plants [5], [6]. Previous models of peaks that occur in succession focused on a ‘temporal-to-spatial’ conversion concept, ‘coordinated by a molecular clock that converts temporal information into a periodic spatial pattern’ [5], [6]. But recently, an experimental study by Dias *et. al* suggested that ‘a clock-and-wavefront’ mechanism is unnecessary for somite formation' [7]. Here we provide a ‘no clock’ model in which spatial peaks appear sequentially in time.

The transverse instability is a Turing-type instability that gives rise to the splitting of the leading activator peak, into 2 simultaneously formed peaks, each of which then becomes the leading activator peak of the daughter branches. When both instabilities are satisfied, a mixed branching pattern of tip splitting and side branching occurs.

## Methods

### Mathematical Model

We used a 4 variable reaction-diffusion PDE based on the work of Meinhardt [4]. The PDE has 4 variables A, H, S, and Y, each of which is a function of space and time. Three represent concentrations of postulated morphogens: an activator A, inhibitor H, and substrate S, while the fourth is a marker for cell differentiation Y.

The equations of the model are:
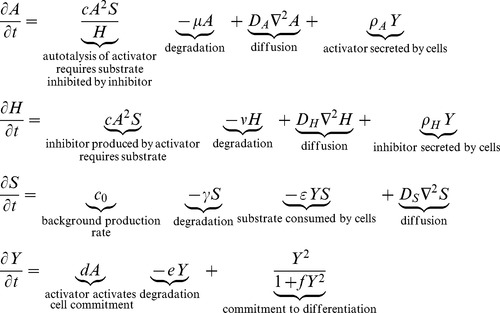
The model assumes that activator A, inhibitor H and substrate S are all diffusible substances, with diffusion coefficients 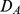, 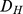 and 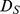, respectively. Activator A is up-regulated by itself in autocatalytic reaction kinetics at rate c (this is the 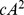 part of the first term in the A-equation) [7]. This autocatalytic process is augmented by substrate S, which is represented by the term 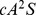. The production of activator A is inhibited by inhibitor H, which is modeled by placing the H term in the denominator (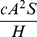 in the A equation). Differentiated Y cells secrete activator A at a rate 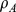 (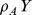
in the A equation). The production of inhibitor H is increased by activator A, again requiring the presence of substrate S (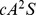 in the H equation). Differentiated Y cells also produced inhibitor H at a rate 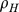 (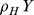 in the H equation). Substrate S is produced at a rate 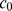, and is consumed by differentiated Y cells at a rate 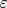. The fact that substrate is consumed by cells in a stoichiometric reaction is modeled by the product term 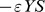 in the S-equation. Cell commitment (Y = 1 means a committed cell) is irreversibly activated when the concentration of activator A grows over a certain threshold, as formulated by the sigmoidal term in the Y equation. A, H, Y and S are all subject to first-order degradation, at rates 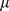, 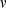, 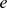, and 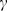, respectively.

### Decoupling the branching process

The 4 variables in the PDE model play distinct roles in the branching process. The interaction between Y (differentiated cells) and S (substrate) produces the spatial extension of the Y-stalk (and the depletion of the substrate at the same time). The dynamics between activator A and inhibitor H are responsible for the local patterns formed on the Y-stalk. Therefore, the branching process can be decoupled into two semi-independent sub processes:

1. Classic activator-inhibitor dynamics (A/H local dynamics)
2. Extension of the Y-stalk (Y/S dynamics)

**Figure 1:**
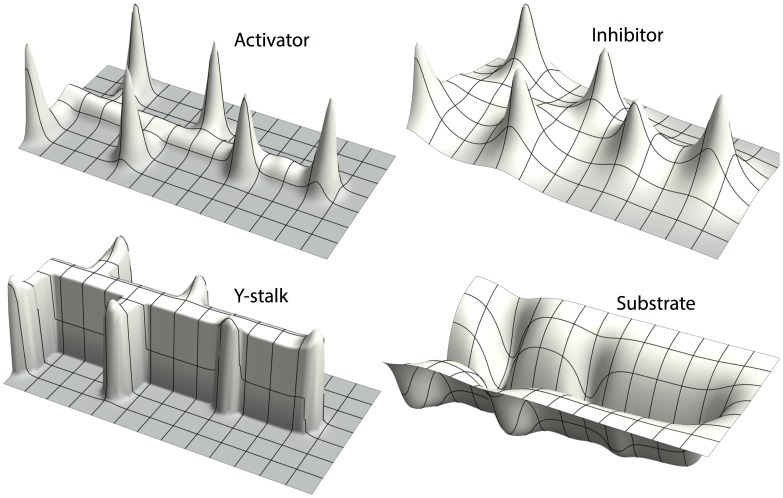
Spatial pattern of each variable in side branching. The spatial pattern of activator A (*top left*) and inhibitor H (*top right*) overlap, while the Y-stalk (*bottom left*) and substrate S (*bottom right*) are spatially complementary. (morphogen concentrations are denoted as z-axis height).

We illustrate the decoupling method in the case of side branching. (Fig. 1)

Note that the spatial patterns of A and H overlap (Fig. 1 *top row*): whenever there is an activator peak, there will also be an inhibitor peak at the same spot. This is because activator produces inhibitor. The inhibitor peaks are flatter and have less sharp boundaries, due to the more rapid diffusion of the inhibitor (Dh>>Da).

The Y/S dynamics produce the extension of the stalk in a three-step process: (1) Y cells are activated to irreversibly differentiate (Y goes from 0 to 1) at sites where activator concentration is high. (2) However, this cell commitment occurs at the expense of consuming local substrate S (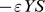 term in S equation), which is required for the maintenance of the activator peak. (3) This substrate depletion drives the activator peak to migrate forward, toward fresh substrate, which in turn drives new cells to commitment. As a result, the spatial pattern of Y is a recording of the path of activator peaks. Commitment pins down and ‘freezes’ the path of activator peaks, and sculpts the substrate S to have a spatially complementary pattern (Fig. 1 *bottom row*).

Therefore, we reduce the 4 variable model by making the Y-stalk and the substrate S to be controllable parameters in the local A/H dynamics. We approach this by setting Y and S to be parameters that are functions of space and time, 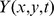 and 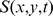, that we designate. Note that they are not part of the PDE iteration. The specific form of the functions 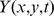 and 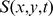 will be provided in the numerical simulation section, and in the figure legends of related simulations, as well as related text.
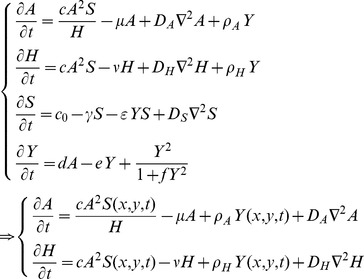



### Strategy of this paper

In this paper, we will use this decomposition into the A/H and Y/S subsystems to analyze branching dynamics. We find that there are two distinct pattern forming instabilities at work in the A/H system, one in the longitudinal direction (the direction of stalk growth) and the other in the direction transverse to the growth axis.

- the longitudinal instability is a time-dependent, nonlinear bifurcation beyond the Turing instability, that is, far from equilibrium.
- the transverse instability is a Turing bifurcation in the A/H subsystem that is produced by increased domain width.

**Figure 2:**
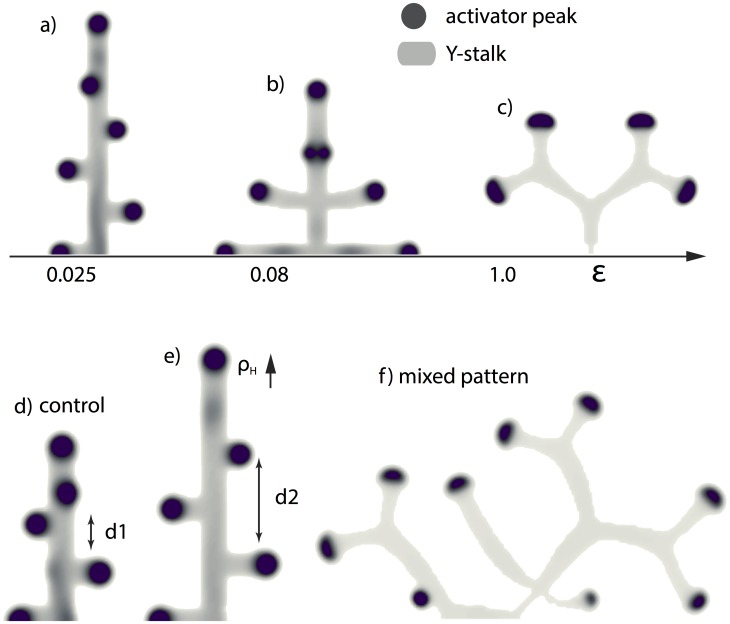
Different branching patterns produced by altering key parameters. (**a, b, c**) as one of the key parameters 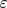 gradually increases, the branching mode produced by the full model changes from (a) side branching with left-right alternating order to (b) side branching with symmetry, and then to (c) tip splitting. 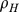 = 0.0001 and 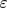 = 0.025/0.08/1.0 from left to right respectively. (**d, e**) as another key parameter 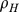 increases, the spatial distance between side branches increased. 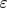 = 0.025, 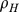 goes from 0.000025 to 0.0002. (**f**) mixed pattern formation of tip splitting and side branching, when 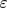 = 0.85, 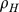 = 0.00003, and and 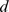 = 0.004. Parameters: 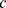 = 0.002, 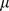 = 0.16, 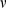 = 0.04, 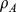 = 0.03, 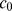 = 0.02, 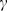 = 0.02, 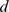 = 0.008, 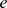 = 0.1, 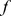 = 10, 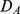 = 0.02, 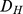 = 0.26, 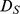 = 0.06.

These two instabilities, working in the longitudinal and transverse directions, are responsible for the main phenomena of branching. We will use these dynamical principles and simulations to address the following questions:

1. One parameter that controls the switch from side to tip branching is the consumption rate of substrate by Y cells, 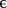 (Fig. 2 a–c). What is the mechanism of this dependence?
2. Several parameters control the spacing interval between side branches, for example, the rate of inhibitor production by differentiated Y cells, 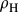. As 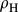 is increased, the spacing interval between side branches increases to the point where no side branching occurs at all (Fig. 2 d, e). Why is this the case?
3. Why does tip splitting occur (Fig. 2 c)?

### Other models of lung branching morphogenesis

Since Meinhardt's 1976 paper, there have been several other mathematical models that study lung branching phenomena. The model of Menshykau *et al*. [8] is based on the reaction and diffusion of FGF10 and SHH as well as the SHH receptor patched (Ptc). They showed that side branching and tip bifurcation can be distinguished by choosing different growth speeds of the lung bud. But in their model, the growth of the lung bud is not caused by the morphogens, instead, it is imposed by a command that the cylinder-shaped lung bud grow as a function of time. Therefore, their model is *not* a model of morphogenetic growth, but rather, a model of periodic spots appearing surrounding the lung bud; they have branching *points* but not actual branching. Thus, Menshykau's paper is not a model for what Clement *et al*. [9] call ‘shape emergence’ or morphological growth.

Clement *et al*. [9], [10] approach branching morphogenesis through diffusion-based mechanisms. Their work addresses the importance of ‘shape emergence’. Their model considered two factors during lung development, the spatial diffusion of FGF10, and the epithelial growth response to an FGF10 gradient. Their simulations showed that different epithelial growth functions could produce branching patterns with different morphological features, including side branching and tip splitting. However, *why* a certain kind of growth function can produce one kind of branching rather than the other was not investigated. Instead, the importance of side branching was dismissed, by saying “It is unsure that side-branching plays a significant role in lung development” [10].

Later on, Clement *et al* extended their diffusion-based model into 3 dimensions [11]. They showed that ‘the self-avoiding branching pattern’ generated by their model is robust in both 2- and 3-dimensional simulations. They also showed that, different growth responses to morphogen gradients (such as linear or sigmoidal functions, similar to their 2-dimensional approach) could generate branching morphologies with different morphological details. However, the fundamental relationship between different types of growth response and different branching patterns was not explained. While Clement *et al* focused on the self-avoidance feature, the issue of side branching vs. tip splitting was not investigated.

On the contrary, the PDE model in our paper [3] includes morphological growth as a causal response to fundamental mechanisms, a differential equation rather than a stipulated function. Therefore, cascades of branching events can naturally emerge from our model or others of this kind. Side branching and tip splitting can be distinguished by altering simple key parameters. The branches also show self-avoidance features.

### Numerical simulation

Our models were numerically simulated using a forward Euler method with no-flux boundary conditions. The spatial domain was discretized into a uniform grid with space step d*x* = 0.3. The domain size for simulations was 200×200. For the diffusion operator, we used a four-point Laplacian. The initial conditions were as follows:

For the 4 variable model, at the beginning of the simulation, activator A, inhibitor H and substrate S are uniformly distributed in space. Activator and inhibitor have very small values: A = 0.001, H = 0.01, while substrate has a high value: *S* = 1.0. For the initial condition of Y, almost all sites are set to Y = 0, except for a small rectangularly-shaped region at the left edge of the simulation boundary.

For the reduced A/H model, at the beginning of the simulation, activator A, and inhibitor H are uniformly distributed in space. Activator and inhibitor have very small values: A = 0.001, H = 0.01. The initial condition of Y and S will differ depending on the experimental setup. Basically, when we are testing the pattern formation of the AH system on a stationary Y stalk, the spatial distributions of Y and S are Y(x,y,t)  = 1.0, S(x,y,t)  = 0.6 inside the stalk, and Y(x,y,t)  = 0.0, S(x,y,t) = 0.0 outside the stalk; when we are testing the pattern formation of the AH system with a growing Y stalk, Y and S will have high values inside the rectangle, and low values outside. The only difference will be that the length of the rectangle grows over time. When we are testing the migration of the activator peaks, we set a high concentration of substrate S(x, y, t)  = 1.0 surrounding the Y stalk. (The spatial distribution of Y and S in different experiments will be addressed in detail in the relevant text or legends.)

Programs were written in CUDA for GPU implementation. 2D contour plots were done in Mathematica. All codes were run on a platform with a CPU from Intel (Model: Intel Core i7–2600), GPU from NVIDIA (Model: NVIDIA GTX580), and 8GB memory. (Code S1)

## Results

### Side branching: the longitudinal instability

#### Periodic activator peaks along the Y-stalk determine pre branch sites (Fig. 3)

**Figure 3:**
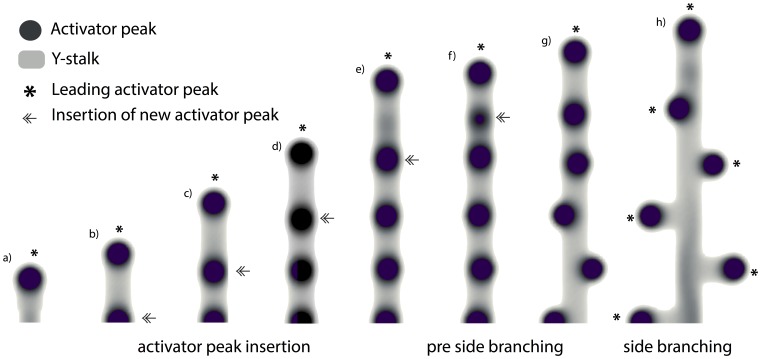
Periodic activator peaks along the Y-stalk determines pre branch sites. (**a**) the leading activator peak emerges at the growing tip, marked by *. (**b**) Forward migration of the leading activator peak produces the elongation of the Y-stalk. When enough space is created behind the leading activator peak, a new activator peak will be induced right behind it, marked by double arrow. (**c–f**) After several rounds of peak insertion, activator peaks line up along the Y-stalk. (**g, h**) later on, these activator peaks migrate out in the transversal direction, each of which becomes the leading activator peak of the newly formed side branches, marked by *. Parameters: 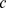 = 0.002, 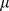 = 0.16, 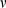 = 0.04, 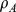 = 0.03, 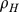 = 0.0001, 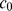 = 0.02, 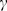 = 0.02, 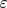 = 0.0025, 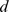 = 0.008, 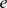 = 0.1, 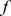 = 10, 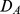 = 0.02, 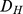 = 0.26, 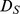 = 0.06.

In side branching, two things happen, in sequence: 1) new activator peaks are periodically inserted on the Y-stalk, always immediately behind the leading activator peak (Fig. 3 a–f). 2). Then, each newly-inserted activator peak migrates outward and gives rise to a side branch (Fig. 3 g, f).

The first activator peak (Fig. 3a), at the tip of Y-stalk, results from inhomogeneous initial conditions (the rectangularly shaped Y-stalk at the boundary of the domain). We call this the leading activator peak (marked by *). It consumes the local substrate, and migrates forward toward fresh substrate. As a result of this migration, the Y-stalk elongates. This creates more space, which enables a new activator peak to be inserted right behind the leading activator peak (Fig 3b, marked by a double arrow). Several rounds of peak insertion result in a periodic pattern of activator peaks along the Y-stalk (Fig 3c–f). These peaks become the source of future side branches.

We then studied how this periodic pattern is created, and the how the spacing between them is controlled.

#### A/H pattern formation along the Y-stalk creates periodic activator peaks

Since periodic activator peaks only exist on the Y-stalk, we asked whether this is due to the profile of Y and S on the stalk. We noted that Y and S concentrations are almost constant along the Y-stalk: Y∼1.0 and S∼0.6 (Fig. S1).

So we asked what pattern would the A/H system form when Y and S are homogeneously distributed in space, S(x,y,t)  = 1.0, and Y(x,y,t)  = 0.6, and the initial conditions of A and H are close to their equilibrium values, with a small (2%) random perturbation. Under these conditions, no pattern formed (Fig. S2 a). This rules out a linear Turing instability, which is further confirmed by the dispersion relation: there is no wavenumber (k) window that has a positive growth rate over time (Fig. S2 b).

**Figure 4:**
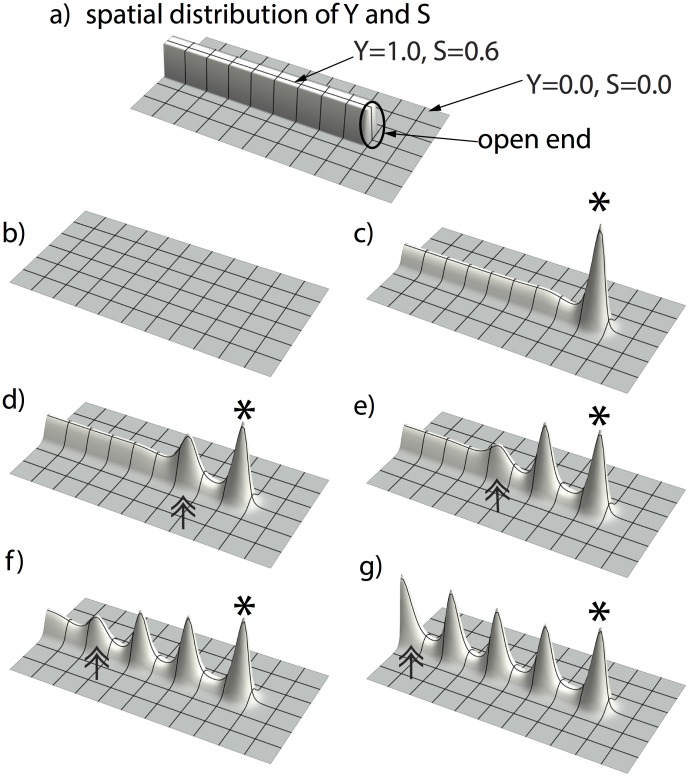
Activator peaks march inward and evolve periodic patterns along the YS domain. (**a**) initial condition of Y and S: high concentrations (Y = 1.0 and S = 0.5) inside the rectangle (5 space steps wide ×80 space steps long) and low concentrations (Y = 0.0 and S = 0.0) outside that rectangle. (**b, c**) a first activator peak emerges at the open end of the rectangularly-shaped YS domain, marked by the asterisk. (**d, e, f, g**) this first activator peak induces new activator peaks to form along the YS domain, marked by double-arrows, in a wave-like manner, until the YS domain is filled up. Parameters: 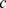 = 0.002, 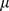 = 0.16, 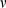 = 0.04, 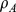 = 0.03, 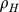 = 0.0001, 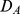 = 0.02, 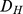 = 0.26.

However, when we set the initial conditions to be inhomogeneous, with S and Y having high concentrations *inside* the rectangle and low concentrations *outside* that rectangle (Fig. 4a), a periodic pattern of activator peaks evolved along the Y-stalk (Fig. 4b–g). Since the initial distribution of the parameters Y and S must be spatially heterogeneous, it is clear that this pattern formation of the A/H subsystem along the Y-stalk is pattern formation far from equilibrium.

#### Appearance of the first activator peak

**Figure 5:**
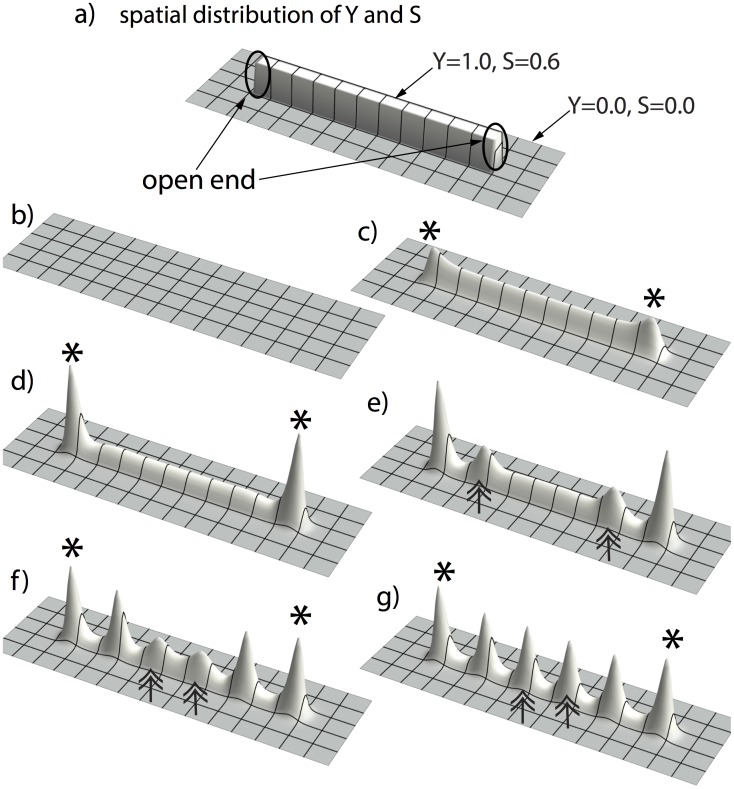
Simulation with YS domain having two open ends. (**a**) initial condition of Y and S: we place the rectangular YS domain in the center, with high concentrations (Y = 1.0 and S = 0.5) inside the rectangle (5 space steps wide ×80 space steps long) and low concentrations (Y = 0.0 and S = 0.0) outside that rectangle. (**b, c**) two activator peaks emerge simultaneously at the two open ends of the YS domain, marked by the asterisks. (**d, e, f, g**) these two activator peaks induce more activator peaks to form along the YS domain, marked by double-arrows, in a wave-like manner, until the YS domain is filled up. Parameters: 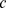 = 0.002, 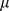 = 0.16, 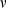 = 0.04, 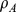 = 0.03, 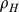 = 0.0001, 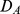 = 0.02, 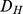 = 0.26. Space step dx = 0.3, time step dt = 0.4dx^2^.

The first activator peak appears at the open end of the rectangularly-shaped YS domain. (Figure 4 a–c). The first activator peak always emerges in the geometry when the Y-stalk is surrounded by a sufficient amount of non Y-stalk, in other words, at the open end of the rectangle (Fig. 4a). This is due to the initial condition on the YS domain: for cells located at the open end, less inhibition is exerted on them because fewer cells are active in the neighborhood (where Y = 0). Thus, the heterogeneous distribution of YS triggers the first activator peak to form. We tested this ‘less inhibition at the open end’ hypothesis by another simulation, in which the YS rectangle was placed in the center of the domain, with two free ends, instead of having one end on the domain boundary (Fig. 5a). Now, *two* activator peaks emerged simultaneously at the two open ends of the rectangle (Fig. 5 b–c).

#### Induction of new activator peaks

**Figure 6:**
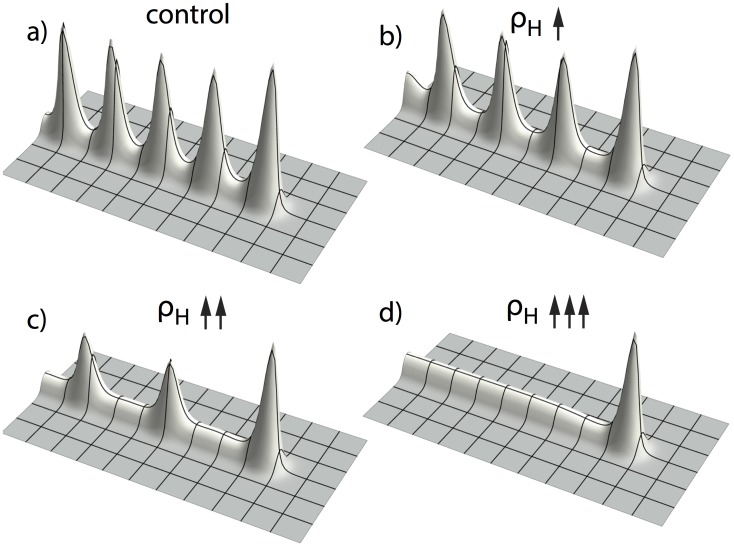
Increased 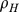, increased spacing between activator peaks. In the simulation of A/H dynamics, with Y and S having high concentrations inside the rectangle (5 space steps wide ×80 space steps long), and low concentrations outside (details see figure 4a). When 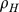 increases, the spacing between activator peaks increased. Parameters: 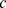 = 0.002, 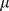 = 0.16, 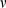 = 0.04, 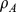 = 0.03, 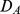 = 0.02, 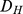 = 0.26, 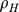 = 0.00005(a), 0.0002(b), 0.00035(c), and 0.0004(d). Space step dx = 0.3, time step dt = 0.4dx^2^.

New peaks then emerge periodically in space and time, marching inward along the stalk (Fig. 5b–g, Fig. 6b–g) in a wave-like manner.

The wave is a response to the large perturbation created by the initial activator peak. This peak also produces inhibitor, which diffuses faster in space, creating an ‘inhibition zone’ for each activator peak, inside which no other activator peak can form. However, beyond that inhibition zone, a new activator peak can be induced if there are sufficient local sources of A to trigger autocatalysis. In the Y-stalk, there are such local sources of A, since Y cells make A at a rate 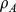. A sufficient local source of A can only happen when Y and S have high values, and is therefore restricted to the Y-stalk. The size of the inhibition zone, hence the spacing interval between activator peaks, can be altered by any parameters that change the A/H dynamics, such as 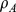 and also 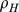, the production rate of inhibitor (Fig. 6). Our simulation shows that when 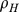 increases, the spatial interval between activator peaks becomes larger (Fig. 6a, b, c), until no pattern formation forms on the Y-stalk at all (Fig. 6d). Since the activator peaks later give rise to side branches, the spatial interval between peaks on the Y-stalk gives the spacing of the side branches (see section below *Migration of activator peaks in the transversal direction*).

The mathematical nature of the instability inducing the secondary peaks is not immediately clear. It is not a simple (linear) Turing instability, since it requires a large perturbation as an initial condition and appears in time as well as space.

### Stalk growth produces insertion of activator peaks

**Figure 7:**
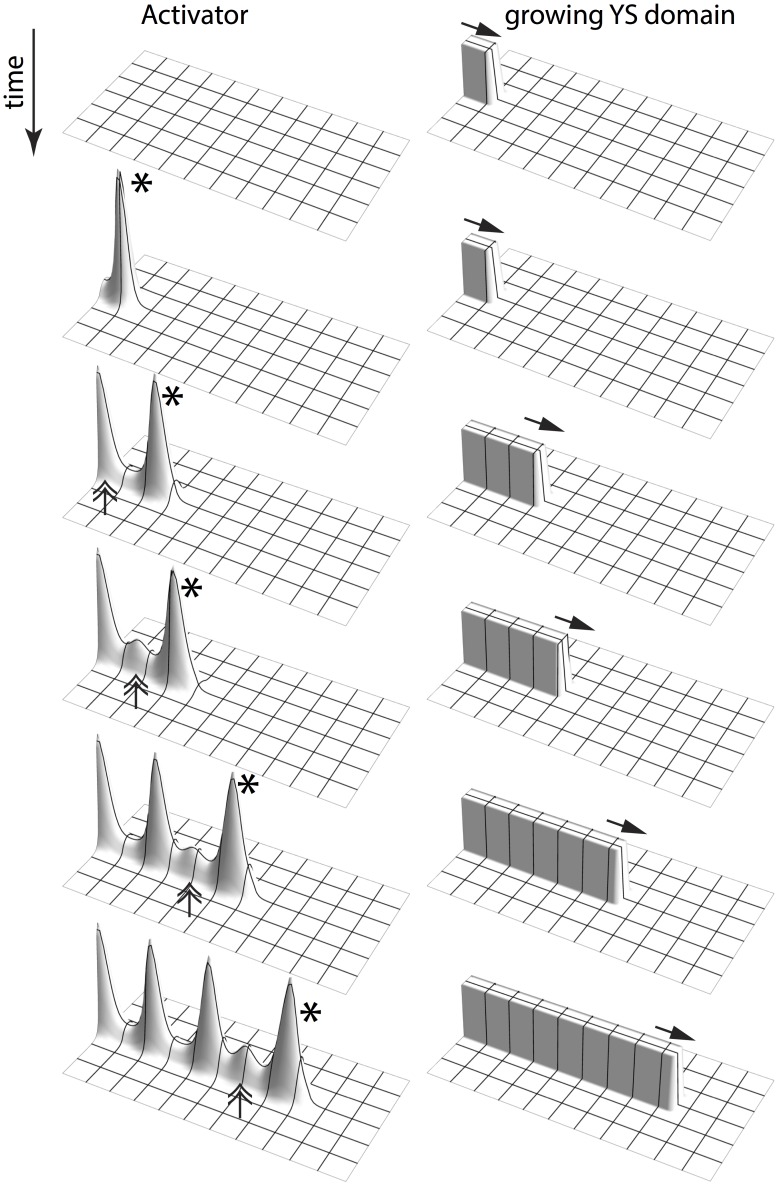
Growing YS domain produces activator peak insertion. When we command the rectangular YS domain to extend over time (by setting the length of the rectangular to be a function of time. The initial length of the rectangular area is 10 space steps. The length increases by 1 space step every 10,000 time steps; the width of the rectangular is held constant at 5 space steps, space step dx = 0.3, and time step dt = 0.4 dx^2^), A/H dynamics forms the activator peak insertion. The left column is the change of activator spatial pattern over time, and the right column is the corresponding spatial pattern of YS domain. Time increases from top to bottom. The first activator peak appears at the open end the YS domain (marked by the asterisk). More activator peaks will be induced and emerge right behind the leading activator peak when the growth creates enough space (marked by double-arrows).

In real branching, the stalk is not stationary. It grows over time. We therefore studied the effects of stalk growth on peak formation. In one experiment, we commanded the rectangular YS domain to extend over time (Fig. 7 *right column*). As expected, the first activator peak emerges on the open end of the Y-talk, due to the geometry of the initial condition (Fig. 7 *first row*). However, the initial length of the YS domain is not enough for a second peak to form, until enough space has been created by growth. Therefore, instead of marching inward, new activator peaks insert immediately behind the leading activator peak. This phenomenon is observed in actual lung branching [3], [12]. So the growth of the YS domain produces the insertion of new activator peaks along the Y-stalk.

**Figure 8:**
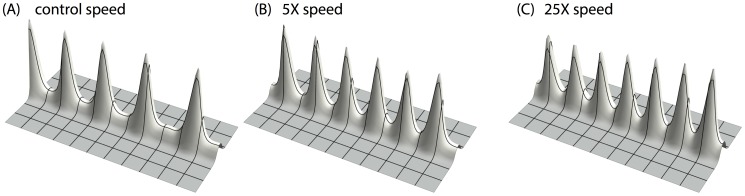
Increased growth speed, increased periodicity of the activator peaks. In the simulation of A/H dynamics with growing YS domain, when we increased the growth speed by a factor of 5 and 25, more activator peaks evolve along the Y-stalk. Parameters: 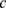 = 0.002, 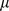 = 0.16, 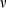 = 0.04, 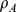 = 0.03, 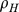 = 0.00005, 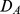 = 0.02, 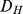 = 0.26. Space step dx = 0.3, time step dt = 0.4dx^2^. The initial shape of the rectangular is 5 space steps wide by 10 space steps long. The speed with which the rectangular extends differs. Control: every 10,000 time steps extend one space grid; 5X: every 2000 time steps extend one space grid; 25X: every 400 time steps extend one space grid.

We also investigated how growth speed influences the pattern formation in the AH system along the Y stalk. By increasing the YS domain growth speed by a factor of 5 and 25 (Fig. 8), we found, somewhat counter-intuitively, that *more* activator peaks evolved along the Y-stalk, with the interval between peaks decreasing. (See Discussion)

#### Migration of activator peaks in the transversal direction

**Figure 9:**
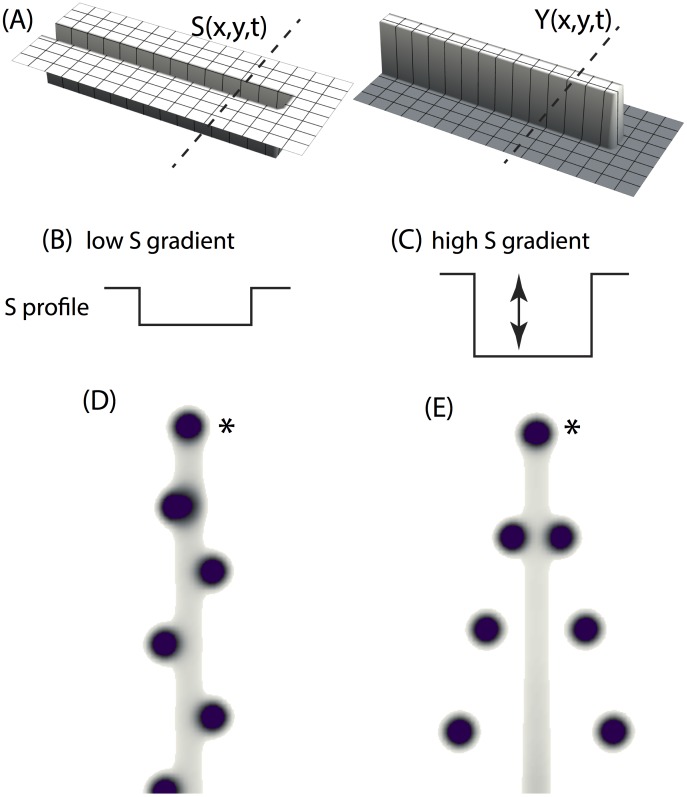
Migration of activator peaks in the transversal direction. (**a**) snapshot of YS domain (the YS domain is growing over time as in figure 7). Morphogen concentration is denoted as z-axis height. Substrate has relatively low values inside the growing rectangle, and relatively high outside the growing rectangle. Y equals to 1.0 inside the rectangle and equal to 0.0 outside that rectangle. The initial rectangular is 5 space steps wide by 10 space steps long. The length of the rectangular increases one space step every 10,000 time steps. Space step dx = 0.3, time step dt = 0.4dx^2^. (**b, c**) profile of S along the dotted line as shown in panel a. The high/low value of S profile is 1.0/0.6 and 1.0/0.4 in panel b and c. Activator peaks migrate out of the YS domain in a left-right order and a symmetrical manner under condition b and c respectively.

Once activator peaks are formed on the Y stalk, they migrate outwards to form side branches, provided there is a significant gradient of substrate (grad S) in the transverse direction (Fig. 9). If the transverse gradient is too small, no outward migration takes place; if it is somewhat larger, outward migration occurs in an alternating left-right style (Fig. 9 b, d), and if the gradient is still larger, peak migration is symmetric (Fig. 9 c, e).

### Tip splitting: the transverse instability

#### Widening of the Y-stalk is required for tip bifurcation

**Figure 10:**
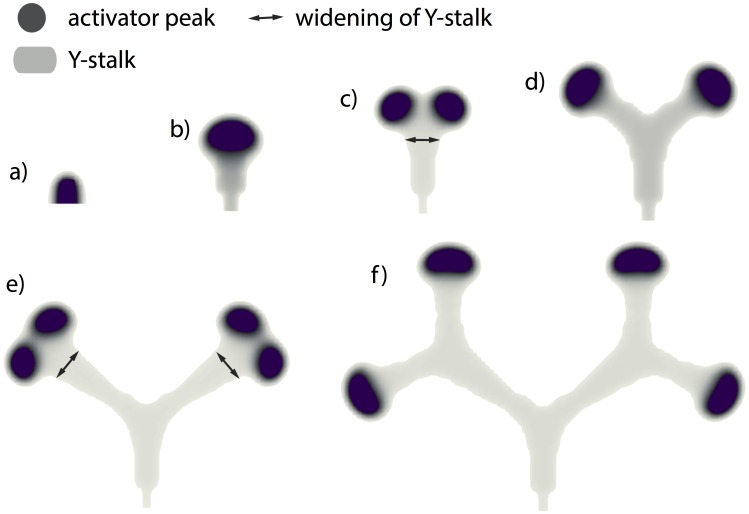
Widening of the Y-stalk is required for tip splitting. (**a**) The first activator peak emerges at the growing tip. (**b**) forward migration of the leading activator peak produces elongation of the Y-stalk. (**c**) the leading activator splits into two daughter peaks as the Y-stalk becomes wider, shown by the arrow. (**d**) each of the daughter activator peaks, from the 1^st^ generation splitting, becomes the leading activator peak of the newly formed stalks. (**e**) 2^nd^ generation of tip splitting occurs when the daughter stalks get wide enough, shown by the arrow. (**f**) each of the granddaughter activator peaks becomes the leading activator peaks of the newly formed stalks, and migrates forward. Parameters: 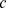 = 0.002, 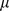 = 0.16, 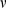 = 0.04, 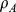 = 0.03, 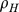 = 0.0001, 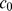 = 0.02, 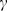 = 0.02, 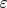 = 1.0, 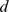 = 0.008, 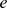 = 0.1, 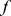 = 10, 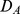 = 0.02, 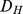 = 0.26, 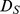 = 0.06.

When epsilon, the consumption rate of substrate by Y cells, increases, the full model produces tip splitting (Fig. 10). Similar to side branching, the first activator peak emerges at the tip triggered by the heterogeneous initial conditions (Fig. 10 a). As we saw, this first activator peak migrates forward toward fresh substrate; Y cells then pin down the path of the activator by irreversibly differentiating (Fig. 10b). However, what distinguishes this case from side branching is that there is almost no substrate left on the Y-stalk, because its consumption rate (the parameter 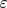) is high. This low substrate level abolishes the ability of the AH subsystem to form periodic patterns on the Y-stalk, so no side branches occur (Fig. 10 c–f).

As the leading activator peak migrates forward, it splits into two, after which Y cells lay down the path of activator peak and form the tip bifurcation (Fig. 10 c). Peak splitting is only seen in the presence of the widening of the Y-stalk (Fig. 10 c, e, shown by the arrows). This led us to suspect that the widening plays a critical role. It is obvious that for activator peak splitting to happen, the domain in the transversal direction must be wide enough to support two activator peaks. The system can accomplish this either by widening the domain, or by shrinking the inhibition zone of the activator peak. The latter requires changing the parameters of the A/H system. This does not happen here, so we conclude that the widening of the stalk is the cause. This was further confirmed by a numerical experiment. When we prevented stalk widening by decreasing the degradation rate of the inhibitor (the parameter 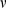), the stalk extended but did not split.

The fact that this is a bifurcation driven by increased domain size L suggests the possibility of a Turing-style transversal instability in the A/H subsystem. Unlike the longitudinal instability, here the two peaks form simultaneously out of the previous peak, and the original peak is not preserved. This phenomenon is well-known in Turing bifurcations, where L/wavelength determines the number of peaks that can be supported. Therefore, we studied the A/H system dynamics as a function of S and Y, to look for conditions that would support a Turing-style size-dependent bifurcation.

**Figure 11:**
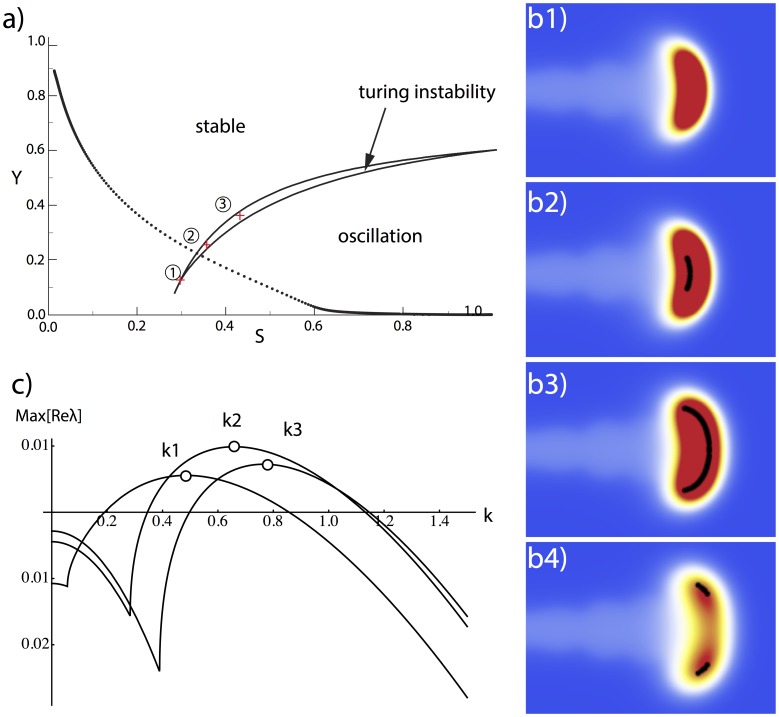
A/H dynamics in tip splitting. (**a**) A/H dynamics as a function of S, Y. When (S, Y) pairs fall into the crescent moon region, the A/H subsystem has a classic Turing instability (by a linear Turing-instability criterion, see [15] page 87). When the (S, Y) pairs are located below the moon region, the temporal behavior of the A/H subsystem is oscillatory. For other (S, Y) pairs, the A/H subsystem has a stable temporal response. The dotted line shows a typical trajectory for cell differentiation. The cell (S, Y) state goes from the bottom right, ‘walks across’ the crescent moon, and reaches the top left. (**b**) Sites of Turing-ready cells formed a strip at the growing tip. When the black strip grew wide enough, it splits into two. Parameters: 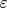 = 2.0, 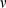 = 0.04, dx = 0.01, dt = 0.4dx^2^, time steps between figures is 5000dt. (**c**) Dispersion relation of k1, k2, and k3 corresponds to the chosen (S, Y) pairs in the crescent moon region numbed 1, 2, and 3, respectively.

Our survey of parameter space revealed that there is indeed a region of (S, Y) parameter space in which the A/H system falls in the Turing instability regime: it is the crescent moon in figure 11 a. Note that for high S and low Y (typical outside the stalk) the A/H system is in the oscillatory regime, below the crescent region. This of course precludes Turing bifurcation, which requires a stable equilibrium in the ODE (ordinary differential equation, when there is no spatial diffusion). But cell differentiation changes Y from 0 to 1, at the expense of depleting substrate S, which moves the system diagonally up and to the left in (S,Y) parameter space. This results in the system crossing the instability boundary into the region of stable equilibria. The crescent moon of parameters that realize the Turing instability lies just inside the stable equilibrium region.

Thus, as each cell develops, it ‘walks’ across parameter space from the lower-right region (oscillatory A/H) through the crescent moon (Turing instability) to the upper left (stable equilibrium but no pattern formation). A typical trajectory is shown by the dotted line in figure 11 a.

We asked where these Turing-ready cells are located in the stalk. We found that they occupy a thin strip along the front of the growing tip (Fig 11 b). Thus the growing tip is continually in the Turing bifurcation regime. All that is required for actual Turing bifurcation to occur, that is, for two peaks to emerge, is that this regime extends over a sufficient length to support the two peaks.

We found that this indeed occurs. As the tip widens, the Turing region (shown in black in figure 11 b1, b2, b3) grows in length until the single peak splits into 2 (Fig. 11 b4).

As a further test of the Turing hypothesis for the transverse instability, we calculated the wavenumber that is predicted by the Turing instability and compared it to the length of the Turing region in the growing tip. This calculation is not exact, because the Turing region at the tip does not have a single value, but represents a distribution of values. We chose 3 (S, Y) pairs in the Turing region, and calculated the wavelengths of the fastest growth modes. These dispersion relations (Fig 11 c) give rough wavenumbers k between 0.4 and 1.0. This yields to a wavelength (2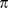/k) of roughly 6–15 space units. We then measured the length of the Turing region (the black stripe in figure 11 b3) and found it to be roughly 6 space units. Therefore, the Turing region is of the right order to produce a length-dependent bifurcation.

## Discussion

We decoupled the branching process of the Meinhardt model into two semi-independent sub processes: A/H dynamics (activator inhibitor interaction) and Y/S dynamics (Y-stalk extension and substrate depletion). We then considered only the A/H dynamics, treating Y and S as parameters that are distributed spatio-temporally. We used this reduced model to explore the mechanisms for side branching and tip splitting.

Our simulation results suggest that side branching results from a longitudinal instability of the A/H subsystem along the Y-stalk, far from equilibrium, while tip splitting is due to a Turing-style instability of the A/H subsystem along the transversal direction, which requires the stalk to be sufficiently wide. The two instabilities do not contradict each other. When both of them are satisfied, tip splitting can coexist with side branching.

We say that the longitudinal instability is nonlinear, and therefore non Turing, because it does not arise spontaneously out of a homogenous initial condition. It requires a large perturbation in the form of an initial stalk. Moreover, it arises sequentially in time, unlike the Turing instability, it arises among cells whose dispersion relation predict no Turing instability (Fig. S1, S2). On the other hand, the transverse instability appears simultaneously over its spatial domain from a homogeneous initial conditions (the black regions in figure 11 b1–4). Moreover this region is exactly the region of cells that are subject to the Turing instability by the eigenvalue analysis.

Our analysis is also very consistent with the results of Crampin *et al*. [13]. Their study investigated “insertion or splitting of concentration peaks” in response to different types of domain growth. In particular, they used *apical* growth (one end) vs. *uniform* growth (both ends). When they coupled Gierer-Meinhardt kinetics to apical growth, new activator peaks inserted behind the moving boundary. This is quite similar to our situation in side branching: when we extend the growth domain of A/H subsystem in the longitudinal direction, new activator peaks emerged right behind the leading activator peak. When Crampin *et al*. used uniform domain growth in their numerical simulation, they saw a splitting of concentration peaks. This is the same as the case in the transverse direction in tip bifurcation, where the stalk widening results the splitting of the leading activator peak into two.

### A/H longitudinal instability vs. temporal-to-spatial conversion

Periodic spatial patterns occur frequently in both animals and plants, for example in animal somitogenesis and in plant root formation. The mechanisms generally proposed for these phenomena often rely on a concept that requires ‘a molecular clock that converts temporal information into a periodic spatial pattern’ [5], [6].

Following this intuition, it might be suggested that the new peaks on the Y-stalk are created by an oscillatory process in the growing tip. Think of a train that is carrying a temporal oscillator. It will create a spatially periodic pattern on the ground, whose spatial wavelength will be the product of the train's speed with the temporal period of the oscillator.

In our model, however, this is not the mechanism of periodic side branching. Along the longitudinal direction, as the stalk grows faster, *more* activator peaks emerge (Fig. 8), not fewer, as would be expected from the oscillator-on-a-train mechanism. The reason why more peaks appear when the growth speeds up is that when the stalk grows faster, this motion physically removes inhibitor behind the leading activator peak: inhibitor becomes locally reduced by physical transport (advection along the stalk), in addition to diffusion and/or degradation. This speeds the removal of inhibitor, allowing additional activator peaks to be inserted. Note that when domain growth is imposed on a system, it plays the role of physical translation of material that is characteristic of advection, as is clearly explained in Crampin *et al*. [13].

As a further confirmation, we followed the temporal behavior at the growing tip, and recorded the change of morphogen concentration over time during growth. No oscillatory processes were detected. Our simulation is also consistent with the results of a recent experimental study, which shows that ‘a clock-and-wavefront mechanism is unnecessary for somite formation’ [14].

## Supporting Information

Figure S1 **Cross section of each variable along the longitudinal growth direction.** We show the 3D plot of each variable (A, H, S, Y), with the morphogen concentration as z-axis height. The bottom panel is the profile of each variable along the elongation direction on the Y-stalk. Y and S values are around 1.0 and 0.6, respectively.
(TIFF)

Figure S2 **No pattern formed in the A/H subsystem when Y, S are spatially homogeneously distributed.** (**a**) When the initial condition of A and H are at equilibrium state with 2% random perturbation (shown as the pepper-and-salt figure on the left), and the distribution of Y and S are homogeneously distributed in space as 1.0 and 0.6 respectively. Simulation results show that the A/H system goes back to equilibrium (the black figure on the right). (**b**) calculated dispersion relation of the A/H system when Y = 1.0 and S = 0.6 indicates that no linear instability exists in this system.
(TIFF)

Code S1 **Numerical simulation CUDA code.** Numerical simulations in this article were written in CUDA for GPU implementation. The Nvidia CUDA Compiler (NVCC) was used to generate to the executable files.
(PDF)
