# Supplementary figures and images for: Mechanisms of Side Branching and Tip Splitting in a Model of Branching Morphogenesis
Source: PLoS One. 2014 Jul 22;9(7):e102718. doi: 10.1371/journal.pone.0102718 (PMC4106868; doi:10.1371/journal.pone.0102718)

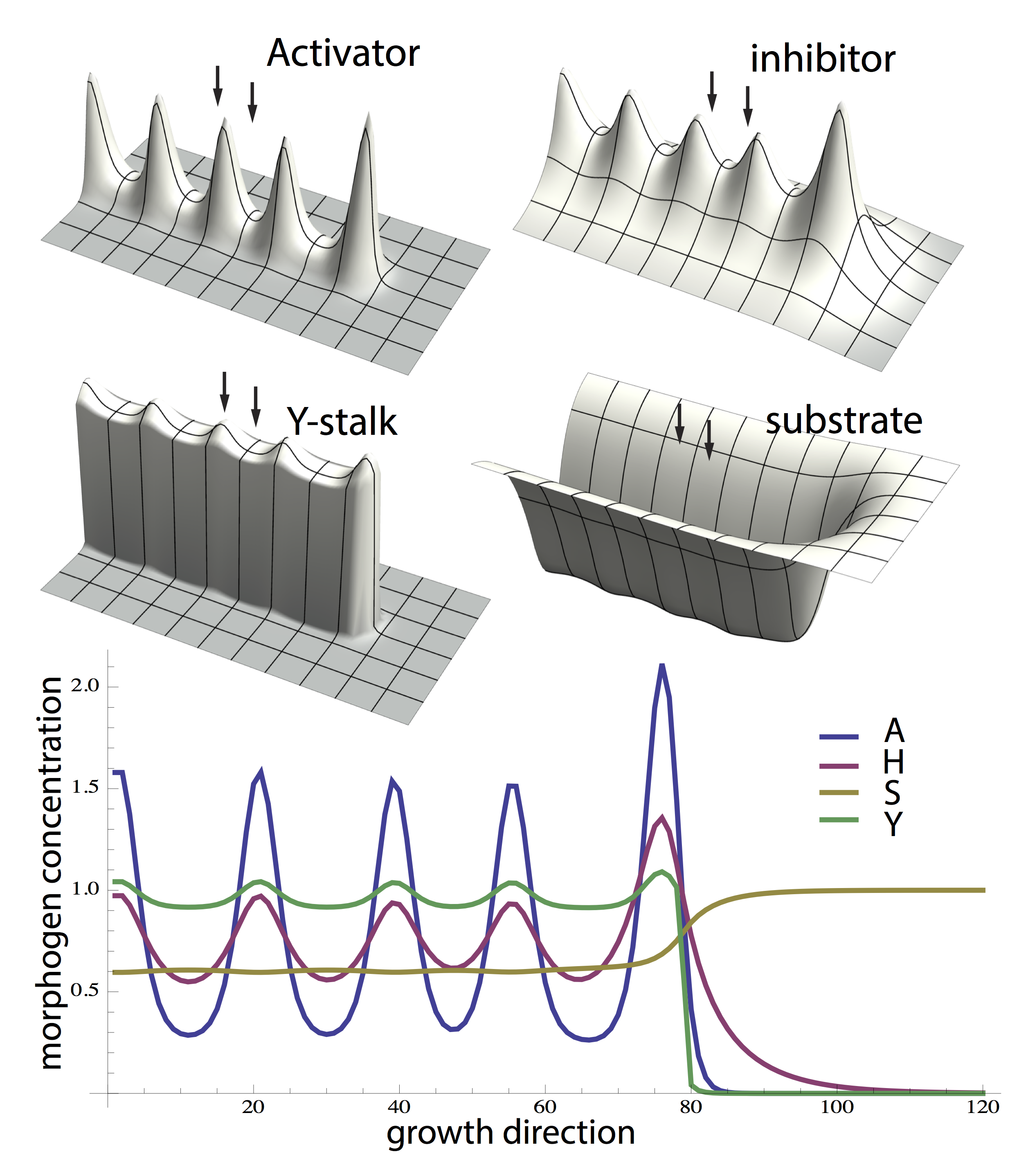

Supplement: Figure S1 — Cross section of each variable along the longitudinal growth direction. We show the 3D plot of each variable (A, H, S, Y), with the morphogen concentration as z-axis height. The bottom panel is the profile of each variable along the elongation direction on the Y-stalk. Y and S values are around 1.0 and 0.6, respectively. (TIFF) [file pone.0102718.s001.tiff]

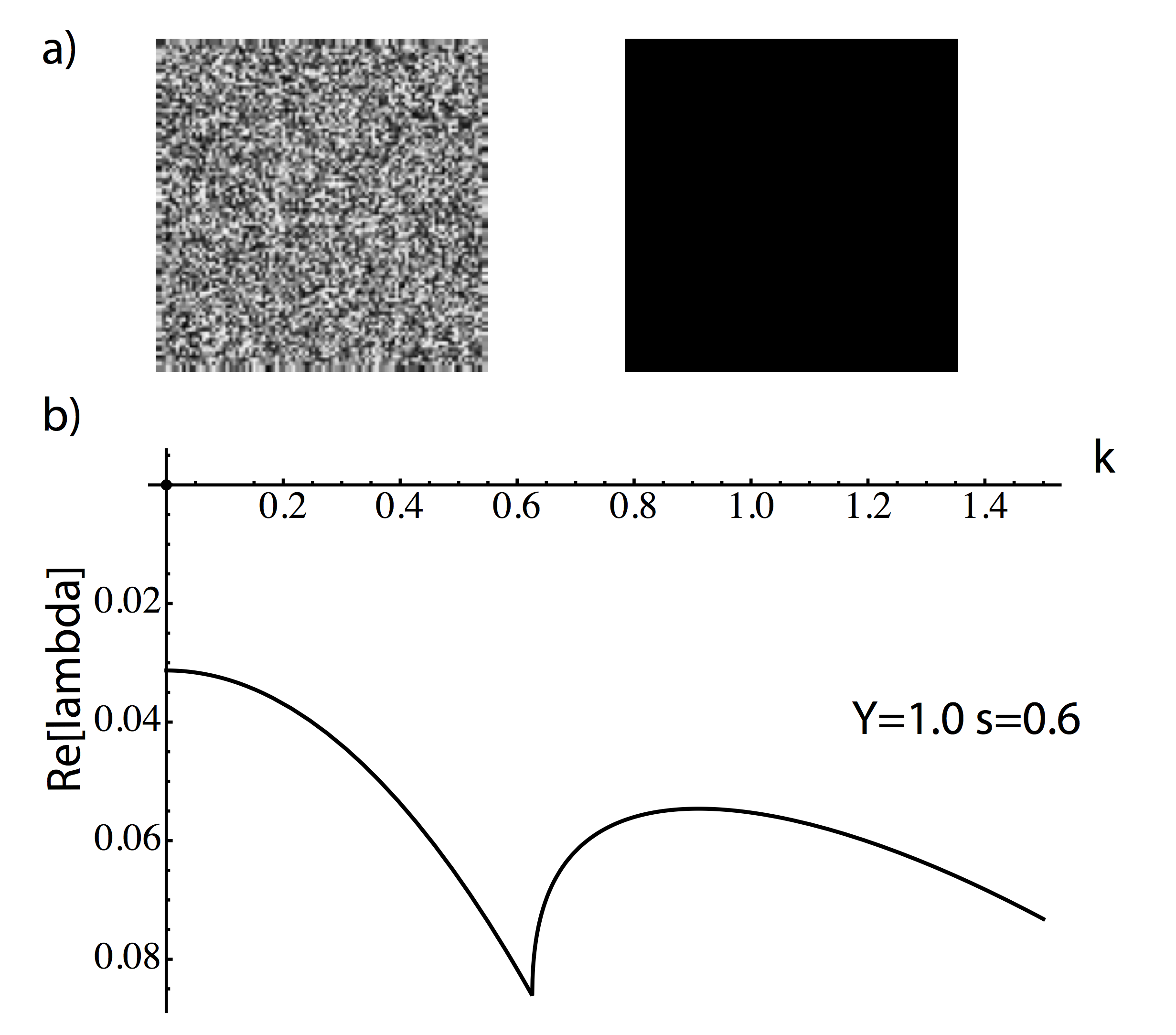

Supplement: Figure S2 — No pattern formed in the A/H subsystem when Y, S are spatially homogeneously distributed. (a) When the initial condition of A and H are at equilibrium state with 2% random perturbation (shown as the pepper-and-salt figure on the left), and the distribution of Y and S are homogeneously distributed in space as 1.0 and 0.6 respectively. Simulation results show that the A/H system goes back to equilibrium (the black figure on the right). (b) calculated dispersion relation of the A/H system when Y = 1.0 and S = 0.6 indicates that no linear instability exists in this system. (TIFF) [file pone.0102718.s002.tiff]
