# Supplementary material for: Mechanisms of Side Branching and Tip Splitting in a Model of Branching Morphogenesis
Source: PLoS One. 2014 Jul 22;9(7):e102718. doi: 10.1371/journal.pone.0102718 (PMC4106868; doi:10.1371/journal.pone.0102718)
Supplement: Code S1 — Numerical simulation CUDA code. Numerical simulations in this article were written in CUDA for GPU implementation. The Nvidia CUDA Compiler (NVCC) was used to generate to the executable files. (PDF) [file pone.0102718.s003.pdf]

```
#include <cstdio>
#include <ctime>
#include <cmath>
#include <cstdlib>
#include <cassert>
#include <numeric>
#include "CImg.h"

///////////////////////////////////////////
#define XLEN              100
#define YLEN              100

#define DELTA_X           0.3
#ifndef D_A
#define D_A               0.02
#endif
#define D_A_2             0.02
#define D_H               0.26
#define D_Y               0
#define D_S               0.06
#define DELTA_TAU         DELTA_X*DELTA_X*0.4

#define A_INIT            0.001
#define H_INIT            0.01
#define Y_INIT            0.001
#define S_INIT            1

/*
#define A_INIT            0.25
#define H_INIT            0.0033
#define Y_INIT            0.02
#define S_INIT            1
*/

#define NUM_1             1
#define NUM_2             2
////////////////// const ////////////////////
// not same //
#ifndef BIO_C
#define  BIO_C            0.002
#endif
#define C_O               0.02
#define  C_2              0.002
// not same //
#define MU                0.16
#define GAMMA             0.02
#define EPSILON           2.0
#define D                 0.008
#define E                 0.1
#define F                 10
```

```
#ifndef RHO_A
#define RHO_A           0.03
#endif

#define RHO_H           0.0001
#define NU              0.04

#ifndef RA1
#define RA1             1
#endif

#ifndef RA2
#define RA2             1
#endif

#define RAND            0
#define RADIUS          2
#define TSTEP           2000

unsigned const jflone = 0x3F800000u;
unsigned const jflmsk = 0x007FFFFFu;

using namespace std;
using namespace cimg_library;

// Grid array

float fga0[XLEN][YLEN];
float fga1[XLEN][YLEN];
int   iga0[XLEN][YLEN];

typedef float ( * mat_t )[YLEN];
__global__ void Init ( mat_t dad, mat_t dhd, mat_t dsd, mat_t dyd ) {
        int i, j;
        i = blockIdx.x * blockDim.x + threadIdx.x;
        j = blockIdx.y * blockDim.y + threadIdx.y;
        if ( i >= 0 && i <= XLEN - 1 && j >= 0 && j <= YLEN - 1 ) {
                dad[i][j] = A_INIT;
                dhd[i][j] = H_INIT;
                dsd[i][j] = 0.0;
                dyd[i][j] = 0.0;
        }
        __syncthreads ( );
        if ( i >= XLEN / 2 - RADIUS && i <= XLEN / 2 + RADIUS && j >=
0 && j <= 10 ) {
                dyd[i][j] = 1.0;
                dsd[i][j] = 0.5;
        }
}
```

```
__global__ void update ( mat_t dad, mat_t dau, mat_t dhd, mat_t dhu,
mat_t dsd, mat_t dsu, mat_t dyd, mat_t dyu, int num) {
        int i, j;
        i = blockIdx.x * blockDim.x + threadIdx.x;
        j = blockIdx.y * blockDim.y + threadIdx.y;
        if ( i <= 0 || i >= XLEN - 1 || j <= 0 || j >= YLEN - 1 )
return;
               // dad -> dau
        // dad -> dau
        dau[i][j] = dad[i][j] + DELTA_TAU * (
                        BIO_C * dad[i][j] * dad[i][j] * dsd[i][j] /
dhd[i][j] -
                        MU * dad[i][j] +
                        D_A * (
                                ( dad[i-1][j] - 2 * dad[i][j] +
dad[i+1][j] ) / DELTA_X / DELTA_X +
                                ( dad[i][j-1] - 2 * dad[i][j] +
dad[i][j+1] ) / DELTA_X / DELTA_X
                              ) +
                        RHO_A * dyd[i][j]
                        );
        // dhd -> dhu
        dhu[i][j] = dhd[i][j] + DELTA_TAU * (
                        BIO_C * dad[i][j] * dad[i][j] * dsd[i][j] -
                        NU * dhd[i][j] +
                        D_H * (
                                ( dhd[i-1][j] - 2 * dhd[i][j] +
dhd[i+1][j] ) / DELTA_X / DELTA_X +
                                ( dhd[i][j-1] - 2 * dhd[i][j] +
dhd[i][j+1] ) / DELTA_X / DELTA_X
                              ) +
                        RHO_H * dyd[i][j]
                        );
        // dsd -> dsu
        // dyd -> dyu
        // growing process
        if ( i >= XLEN / 2 - RADIUS && i <= XLEN / 2 + RADIUS && j ==
num ) {
                dyd[i][num] = 1.0;
                dsd[i][num] = 0.5;
        } else {
                dsu[i][j] = dsd[i][j];
                dyu[i][j] = dyd[i][j];
        }
        __syncthreads ( );
        if ( i == 1 && j == 1 )  {
                dau[0][0] = dau[1][0] = dau[0][1] = dau[1][1];
                dhu[0][0] = dhu[1][0] = dhu[0][1] = dhu[1][1];
                dsu[0][0] = dsu[1][0] = dsu[0][1] = dsu[1][1];
```

```
                dyu[0][0] = dyu[1][0] = dyu[0][1] = dyu[1][1];
        } else if ( i == 1 && j == YLEN - 2 ) {
                dau[0][YLEN-1] = dau[1][YLEN-1] = dau[0][YLEN-2] =
dau[1][YLEN-2];
                dhu[0][YLEN-1] = dhu[1][YLEN-1] = dhu[0][YLEN-2] =
dhu[1][YLEN-2];
                dsu[0][YLEN-1] = dsu[1][YLEN-1] = dsu[0][YLEN-2] =
dsu[1][YLEN-2];
                dyu[0][YLEN-1] = dyu[1][YLEN-1] = dyu[0][YLEN-2] =
dyu[1][YLEN-2];
        } else if ( i == XLEN - 2 && j == 1 ) {
                dau[XLEN-1][0] = dau[XLEN-2][0] = dau[XLEN-1][1] =
dau[XLEN-2][1];
                dhu[XLEN-1][0] = dhu[XLEN-2][0] = dhu[XLEN-1][1] =
dhu[XLEN-2][1];
                dsu[XLEN-1][0] = dsu[XLEN-2][0] = dsu[XLEN-1][1] =
dsu[XLEN-2][1];
                dyu[XLEN-1][0] = dyu[XLEN-2][0] = dyu[XLEN-1][1] =
dyu[XLEN-2][1];
        } else if ( i == XLEN - 2 && j == YLEN - 2 ) {
                dau[XLEN-1][YLEN-1] = dau[XLEN-2][YLEN-1] =
dau[XLEN-1][YLEN-2] = dau[XLEN-2][YLEN-2];
                dhu[XLEN-1][YLEN-1] = dhu[XLEN-2][YLEN-1] =
dhu[XLEN-1][YLEN-2] = dhu[XLEN-2][YLEN-2];
                dsu[XLEN-1][YLEN-1] = dsu[XLEN-2][YLEN-1] =
dsu[XLEN-1][YLEN-2] = dsu[XLEN-2][YLEN-2];
                dyu[XLEN-1][YLEN-1] = dyu[XLEN-2][YLEN-1] =
dyu[XLEN-1][YLEN-2] = dyu[XLEN-2][YLEN-2];
        } else if ( i == 1 || j == 1 || i == XLEN - 2 || j == YLEN -
2 ) {
                 if ( i == 1 ) {
                        dau[0][j] = dau[1][j];
                        dhu[0][j] = dhu[1][j];
                        dsu[0][j] = dsu[1][j];
                        dyu[0][j] = dyu[1][j];
                 } else if ( j == 1 ) {
                        dau[i][0] = dau[i][1];
                        dhu[i][0] = dhu[i][1];
                        dsu[i][0] = dsu[i][1];
                        dyu[i][0] = dyu[i][1];
                 } else if ( i == XLEN - 2 ) {
                        dau[XLEN-1][j] = dau[XLEN-2][j];
                        dhu[XLEN-1][j] = dhu[XLEN-2][j];
                        dsu[XLEN-1][j] = dsu[XLEN-2][j];
                        dyu[XLEN-1][j] = dyu[XLEN-2][j];
                 } else if ( j == YLEN - 2 ) {
                        dau[i][YLEN-1] = dau[i][YLEN-2];
                        dhu[i][YLEN-1] = dhu[i][YLEN-2];
                        dsu[i][YLEN-1] = dsu[i][YLEN-2];
                        dyu[i][YLEN-1] = dyu[i][YLEN-2];
```

```
                    }
            }
    }

    void cout_result ( int n, float ( *dp )[YLEN], const char * type ) {
            float min, max;
            float * d = &dp[0][0];
            float * de = d + XLEN * YLEN;
            char file[256];
            int i, j;
            min = max = dp[0][0];
            for ( ++d; d != de; ++d ) {
                    if ( min > *d ) min = *d;
                    if ( max < *d ) max = *d;
            }
            sprintf ( file, "%s_%.3d.bmp", type, n );
            printf ("%s  %.8lf  %.8lf \n\n", file, min, max );
            float tran_factor = ( max - min ) / 255;
            CImg < unsigned char > img ( XLEN, YLEN );
            for( i = 0; i < XLEN; ++i ) {
                    for ( j = 0; j < YLEN; ++j ) {
                             int b = ( max - dp[i][j] ) / tran_factor;
                            img ( i , j ) = b;
                    }
            }
            img.save_bmp ( file );
            // write to file
            sprintf ( file, "%s_%.3d.txt", type, n );
            FILE * f = fopen ( file, "w" );
            fwrite ( dp, sizeof ( float ), XLEN * YLEN, f );
            fclose ( f );
    }

    void min_max ( float *d, float &min, float &max, float & tran_factor )
    {
            float *de = d + XLEN * YLEN;
            min = max = *d;
            for ( ++d; d != de; ++d ) {
                    if ( min > *d ) min = *d;
                    if ( max < *d ) max = *d;
            }
            tran_factor = ( max - min ) / 255;
    }
    void bi_cout_result ( int n, float ( *dp )[YLEN], float ( *dq )[YLEN],
    const char * type ) {
            int i, j, b;
            char file[256];
            float min_dp, min_dq, max;
            float tran_factor_1, tran_factor_2;
            min_max ( dp[0], min_dp, max, tran_factor_1 );
```

```
        min_max ( dq[0], min_dq, max, tran_factor_2 );
        sprintf ( file, "%s_%.3d.bmp", type, n );
        CImg < unsigned char > img ( XLEN, YLEN, 1, 3 );
        for( i = 0; i < XLEN; ++i ) {
                for ( j = 0; j < YLEN; ++j ) {
                         b = ( dp[i][j] - min_dp ) / tran_factor_1;
                        img ( i, j, 0, 0 ) = b;
                         if ( b < 50  ) {
                                b = ( dq[i][j] - min_dq ) /
tran_factor_2;
                                img ( i, j, 0, 1 ) = b;
                         } else
                                img ( i, j, 0, 1 ) = 0;
                        img ( i, j, 0, 2 ) = 0;
                }
        }
        img.save_bmp ( file );
}
int main ( int argc, char **argv ) {
        long long TAU = 200000;

        clock_t tb, te;
        tb = clock ( );

        float ( * a   )[YLEN] = fga0 ;
        int   ( * rad )[YLEN] = iga0 ;
        int BLOCK_SIZE = 16;

        int memsize = XLEN * YLEN * sizeof ( float ) ;
        // init d_a_down d_h_down d_s_down d_y_down
        float ( * dad )[YLEN];
        cudaMalloc ( ( void ** )&dad, memsize );
        float ( * dau )[YLEN];
        cudaMalloc ( ( void ** )&dau, memsize );
        float ( * dhd )[YLEN];
        cudaMalloc ( ( void ** )&dhd, memsize );
        float ( * dhu )[YLEN];
        cudaMalloc ( ( void ** )&dhu, memsize );
        float ( * dsd )[YLEN];
        cudaMalloc ( ( void ** )&dsd, memsize );
        float ( * dsu )[YLEN];
        cudaMalloc ( ( void ** )&dsu, memsize );
        float ( * dyd )[YLEN];
        cudaMalloc ( ( void ** )&dyd, memsize );
        float ( * dyu )[YLEN];
        cudaMalloc ( ( void ** )&dyu, memsize );
        float ( * gtmp )[YLEN];

        dim3 dimBlock ( BLOCK_SIZE, BLOCK_SIZE );
        dim3 dimGrid  ( ( XLEN + BLOCK_SIZE - 1 ) / BLOCK_SIZE, ( YLEN
```

```
+ BLOCK_SIZE - 1 ) / BLOCK_SIZE );
        // Init
        Init <<< dimGrid, dimBlock >>> ( dad, dhd, dsd, dyd );
        // update
        for ( int k = 0; k <= TAU; ++k ) {
                update <<< dimGrid, dimBlock >>> ( dad, dau, dhd,
dhu, dsd, dsu, dyd, dyu, k / TSTEP );
                gtmp = dad; dad = dau; dau = gtmp;
                gtmp = dhd; dhd = dhu; dhu = gtmp;
                gtmp = dsd; dsd = dsu; dsu = gtmp;
                gtmp = dyd; dyd = dyu; dyu = gtmp;
                if ( k % TSTEP ) continue;
                cudaMemcpy ( a, dau, XLEN * YLEN * sizeof ( float ),
cudaMemcpyDeviceToHost );
                cout_result ( k / TSTEP, a, "a" );
                cudaMemcpy ( a, dhu, XLEN * YLEN * sizeof ( float ),
cudaMemcpyDeviceToHost );
                cout_result ( k / TSTEP, a, "h" );
                cudaMemcpy ( a, dyu, XLEN * YLEN * sizeof ( float ),
cudaMemcpyDeviceToHost );
                cout_result ( k / TSTEP, a, "y" );
                cudaMemcpy ( a, dsu, XLEN * YLEN * sizeof ( float ),
cudaMemcpyDeviceToHost );
                cout_result ( k / TSTEP, a, "s" );
        }
        cudaFree ( dad );
        cudaFree ( dau );
        cudaFree ( dhd );
        cudaFree ( dhu );
        cudaFree ( dsd );
        cudaFree ( dsu );
        cudaFree ( dyd );
        cudaFree ( dyu );
        te = clock ( );
        printf ("%lf\n", double ( te -tb ) / CLOCKS_PER_SEC );
        return 0;
}
```
